# Glucose intolerance after chronic stress is related with downregulated PPAR-γ in adipose tissue

**DOI:** 10.1186/s12933-016-0433-2

**Published:** 2016-08-19

**Authors:** Vitor H. Pereira, Fernanda Marques, Vânia Lages, Filipa G. Pereira, Alexandre Patchev, Osborne F. X. Almeida, Joana Almeida-Palha, Nuno Sousa, João J. Cerqueira

**Affiliations:** 1 Life and Health Sciences Research Institute (ICVS), School of Health Sciences, University of Minho, Campus de Gualtar, 4710-057 Braga, Portugal; 2 ICVS/3B’s-PT Government Associate Laboratory, Braga/Guimarães, Portugal; 3 Max-Planck-Institute of Psychiatry, Munich, Germany

**Keywords:** Chronic unpredictable stress, Glucose intolerance, Metabolic syndrome, PPAR, Lipocalin-2, White adipose tissue

## Abstract

**Background:**

Chronic stress is associated with increased risk of glucose intolerance and cardiovascular diseases, albeit through undefined mechanisms. With the aim of gaining insights into the latter, this study examined the metabolic profile of young adult male rats that were exposed to chronic unpredictable stress.

**Methods:**

Young adult male rats were submitted to 4 weeks of chronic unpredictable stress and allowed to recover for 5 weeks. An extensive analysis including of morphologic, biochemical and molecular parameters was carried out both after chronic unpredictable stress and after recovery from stress.

**Results:**

After 28 days of chronic unpredictable stress (CUS) the animals submitted to this protocol displayed less weight gain than control animals. After 5 weeks of recovery the weight gain rebounded to similar values of controls. In addition, following CUS, fasting insulin levels were increased and were accompanied by signs of impaired glucose tolerance and elevated serum corticosteroid levels. This biochemical profile persisted into the post-stress recovery period, despite the restoration of baseline corticosteroid levels. The mRNA expression levels of peroxisome proliferator-activated receptor (PPAR)-γ and lipocalin-2 in white adipose tissue were, respectively, down- and up-regulated.

**Conclusions:**

Reduction of PPAR-γ expression and generation of a pro-inflammatory environment by increased lipocalin-2 expression in white adipose tissue may contribute to stress-induced glucose intolerance.

## Background

Stress is a state of threatened homeostasis that triggers a spectrum of adaptive responses to re-establish homeostasis [1]. While initially essential for survival, the physiological responses to stress are detrimental to health when activated over a prolonged period. Epidemiological studies show that chronic stress increases the risk of diabetes and metabolic syndrome [2]. However, the biological mechanisms underlying these associations are still not well understood.

Increases in corticosteroid secretion have long been considered to have a causal role in glucose intolerance associated with stress [3, 4]. These hormones enhance hepatic gluconeogenesis, inhibit the secretion and action of insulin, promote differentiation and proliferation of adipocytes, redistribution of fat, and decrease lipoprotein–lipase activity [5, 6]. However, other mechanisms beyond the direct effects of glucocorticoids are implicated in the pathophysiology of glucose intolerance (for review see [7]). The overactivation of the sympathetic system and the promotion of a pro-inflammatory state in the adipose tissue [8] are common features of insulin resistance [9–12]. Adipose tissue inflammation is characterized by macrophage infiltration and expression of pro-inflammatory mediators such as lipocalin-2 (*Lcn2*) [13], tumor necrosis factor-α (TNF-α), interleukin (*Il*)-*1*, *Il*-*6*, and chemoattractant molecules, such as monocyte chemoattractant protein-1 (*Mcp*-*1*) [14]).

In fact, adipocytes are important players in energy expenditure and endocrine homeostasis [15]. In the center of this regulation is the proliferation-activated receptor (PPAR)-γ, a nuclear receptor produced mainly by adipocytes [16]. When activated, PPAR-γ up-regulates the transcription of genes mainly involved in fatty acid metabolism and triglyceride storage, promoting adipogenesis and lipids uptake to the adipose tissue [17, 18] improving whole body insulin sensitivity.

This study aims to unveil biological markers that establish the molecular basis of stress-induced glucose intolerance, with a special emphasis on the visceral white adipose tissue (vWAT). We extended our analysis to a period of recovery following chronic exposure to stress, an aspect often overlooked although of potential importance in understanding how prior insults may program susceptibility to disease.

## Research design and methods

### Animals

Wistar Han male rats (Charles River Laboratories, Barcelona, Spain), aged 2 months at arrival, were used. To avoid the stress of single housing, animals were housed in pairs, under standard laboratory conditions: artificial light–dark cycle of 12 h (lights from 8:00 a.m. to 8:00 p.m.) in a temperature—(22 °C) and humidity (55 %) controlled room; animals had ad libitum access to food (3 % of calories from lipids, diet 4RF21 GLP, Mucedola, Italy) and water.

### Experimental design and chronic stress protocol

**Fig. 1:**
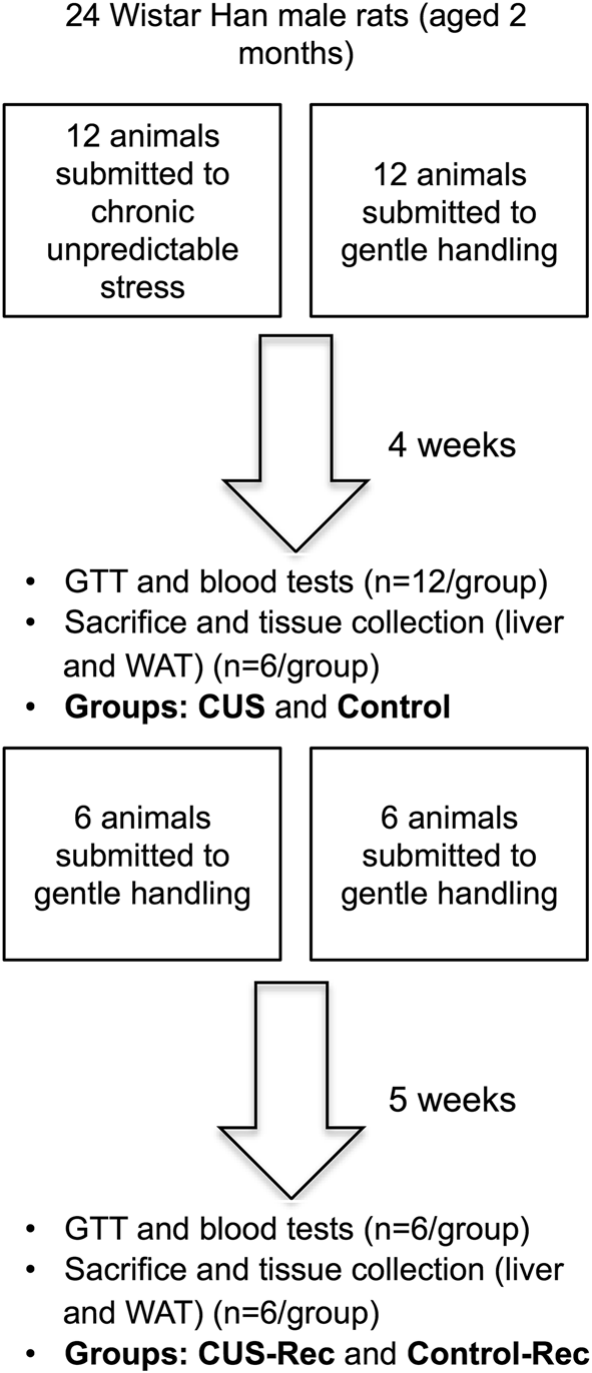
Experimental design of the study. Twenty four animals were equally distributed in four groups: control, CUS; control-Rec and CUS-Rec. CUS and CUS-Rec were submitted to a protocol of chronic unpredictable stress. Animals allocated to control and CUS group were sacrificed after the chronic stress protocol period (28 days) in fasting conditions. The control-Rec and CUS-Rec were sacrificed after a period of 5 weeks post the stress protocol. *CUS* chronic unpredictable stress; *Rec* recovery; *GTT* glucose tolerance test; *WAT* white adipose tissue

Figure 1 depicts the experimental design followed in this study. Briefly, weight-matched animals (n = 24) were distributed equally between experimental and control groups. Animals assigned to the experimental group (n = 12) were submitted to chronic unpredictable stress (CUS) over 28 consecutive days. Six animals from each group were sacrificed (groups CUS and control) after this period and the remaining were sacrificed after 5 additional weeks (groups CUS-Rec and Control-Rec). The protocol of CUS consisted of twice-daily random exposure to one of the following stressors: restraint (1 h), exposition to a hot air jet (30 min), overcrowding (1 h), strobe lights (1 h), shaking (30 min), cold water (15 °C, 1 h) and noise (1 h). A glucose tolerance test (GTT) was performed on the morning after the end of the stress protocol (at 09:00 a.m.). The animals were fasted overnight (12 h) to ensure uniform nutritional condition. Before the intraperitoneal injection of glucose, blood was collected by tail venipuncture for biochemical analysis (hormonal measurements). Six animals from each group were sacrificed 48 h after the GTT so that further analyses would not be influenced by the glucose load or by the acute stress associated with the GTT. Again, the animals were fasted overnight and their visceral WAT (vWAT) and livers dissected out and snap-frozen for molecular analysis (Liver and vWAT). The remaining animals (n = 6 per group) were allowed to recover from CUS for 5 weeks (“recovery period”; group CUS-Rec). After this period a new GTT was performed and the blood collected as previously described. After 2 days, the remaining animals were sacrificed and tissues collected as before. All the blood was collected in conscious animals. Control and animals in “recovery” were submitted to gentle handling (approximately 20–30 min) on a daily basis.

### Glucose tolerance test

The GTT was performed after CUS and after stress recovery following 12 h of fasting as previously mentioned. Glucose was intraperitoneally administered using a 30 % solution of glucose in water at a dose of 200 mg/g of weight. The blood was obtained by puncture of the tail vein and whole blood glucose levels were measured using a hand-held glucometer (Optium Xceed, Abbot, USA) to minimize the volume of blood needed. The glucose was measured at baseline and at 20, 40, 60 and 120 min after the intraperitoneal injection.

### qRT-PCR

Total RNA was extracted from frozen liver and adipose tissue, using TRIzol reagent (Invitrogen Corp, Carlsbad, CA, USA). Then, after quantification in the NanoDrop^®^, 500 ng of total RNA from each sample RNA was reverse-transcribed into first strand cDNA using iScript™ cDNA Synthesis Kit, as described in the manufacturer’s protocol (Bio-Rad Laboratories, Hercules, CA, USA). Quantitative real-time polymerase chain reaction (qRT-PCR) assays were carried out to measure the expression levels of PPAR-γ, PPAR-β, PPAR-α, Lcn2, Mcp-1, IL-1, TNF-α, Cxcl-1, Cxcl-10 and LPL mRNA transcripts. Relative expression levels of mRNAs were calculated by the comparative threshold cycle method using hypoxanthine guanine phosphoribosyl transferase (Hprt) as an internal control (housekeeping gene), after confirming that expression of the gene was not influenced by the experimental conditions.

Primer3 software was used to design the oligonucleotide primers for PPAR-γ (Fw: 5′-gagtttctgaccggactgtgtg-3′; Rw: 5′-aagttggtgggccagaatgg-3′), PPAR-β, PPAR-α(Fw: 5′-aatgctctcgaactaga-3′, Rw: 5′-gcacaatcccctcctgcaac-3′), Lcn2 (Fw: 5′-tcaccctgtacggaagaacc-3′; Rw 5′-tcggtgggaacagagaaaac-3′), Mcp-1 (Fw: 5′-tagcatccacgtgctgtctc-3′; Rw: 5′-tgctgctggtgattctcttg-3′), IL-1 (Fw: 5′-ggcttccttgtgcaagtgtc-3′; Rw: 5′-tgtcgagatgctgctgtgag-3′), TNF-α (Fw: 5′-atgggctccctctcatcagt-3′; Rw: 5′-gcttggtggtttgctacgac-3′), Cxcl-1 (Fw: 5′-ggcttccttgtgcaagtgtc-3′; Rw:5′-tgtcgagatgctgctgtgag-3′), Cxcl-10 (Fw: 5′-ggcttccttgtgcaagtgtc-3′; Rw: 5′-tgtcgagatgctgctgtgag-3′), LPL (Fw: 5′-agaacgcatcatccgaagac-3′; Rw: 5′-tgctcacactcacgttcaca-3′) and Hprt (Fw: 5′-gcagactttgctttccttgg-3′; Rw: 5′-tccactttcgctgatgacac-3′). qRT-PCR was performed on a CFX 96TM real time instrument (Bio-Rad), using QuantiTect SYBR Green RT-PCR reagent kit (Qiagen, Hamburg, Germany), according to the manufacturer’s instructions, using equal amounts of RNA from each sample. Product fluorescence was detected at the end of the elongation cycle. A single sharp peak was exhibited in all melting curves at a temperature characteristic of the primers used.

### Immunohistochemistry

Animals were deeply anaesthetized with pentobarbital and transcardially perfused with saline before excision of vWAT surrounding the mesenterium; tissues were immersed in paraformaldehyde, before embedding in paraffin. Subsequently 4 µm sections were cut and stained for Lcn2 by immunohistochemistry, as described elsewhere [19]. Briefly, sections were deparaffinized and rehydrated prior to antigen retrieval with citrate buffer 10 mM. Endogenous peroxidases were blocked using 3 % hydrogen peroxide in water for 30 min before probing with primary antibody, anti-mouse lipocalin-2/neutrophil gelatinase-associated lipocalin (LCN2/NGAL) (1:400; R&D Systems, Minneapolis, MN, USA), diluted in PBS containing 0.3 % Triton X-100 (PBS-T) and 0.4 % bovine serum albumin (Sigma, St. Louis, MO). Thereafter, sections were incubated with biotinylated anti-goat secondary antibody and treated with streptavidin peroxidase conjugate (ABC kit; Sigma), before being developed with 3,3′-diaminobenzidine tetrahydrochloride hydrate (DAB; Sigma) and counterstaining with hematoxylin-eosin. An Optical (BX61, Olympus, Hamburg, Germany) microscope was used to analyze the samples. Average adipocytes density and volume were assessed using StereoInvestigator^®^ software (MicroBrightField, Williston, VT, USA). Two sections from each animal (n = 5/group) were analyzed (five different fields/section). The area (µm^2^) of these fields was estimated using planimetry and adipocytes were counted manually, using the fractionator probe. Individual adipose tissue densities were determined from the mean density in each frame (10/animal). The average volume of the adipocyte was assessed using the nucleator probe, a software tool to estimate the mean cell volume of a population, as described elsewhere [20]. The center of the largest cell, in all ten fields analyzed from the two sections, was selected and five isotropic rays were generated, proceeding outward, and the place where they intersected the cell’s limit was selected. The average adipocyte volume, of each animal, was determined as the mean of the volume of all the adipocytes measured (approximately 10/animal).

### Hormones

Serum was frozen at −80 °C until further processing. ELISA assay kits were used to measure insulin (Mercodia, Sweden), leptin (Merck Millipore, Darmstadt, Germany) and adiponectin (Merck Millipore). Corticosterone was measured using a radioimmunoassay assay (MP Biomedicals, Santa Ana, CA, USA). Cholesterol was measured by an enzymatic-colorimetric assay (Spinreact, Girona, Spain). All procedures were performed following the recommendations of the manufacturers. The blood used to perform these measurements was collected at the time 0 of the GTT and after a period of 12 h of fasting.

### Statistical analysis

Results are expressed as mean ± standard deviation of the mean. A repeated measures analysis of variance (ANOVA) was performed to determine the effects of the variables ‘time’ and ‘group’ in both weight and GTT. A Bonferroni post hoc test was performed whenever significant interactions were found. A two-tailed Student’s t test was used to compare means of each experimental group with the respective control group when applied. Real time PCR results are expressed in fold-change compared with the control group at that the respective time-point. All analyses were performed using *Statistical Pack for Social Sciences* v.22 (SPSS) and Prism 6.

## Results

### Stress induces a decrease in body weight gain without altering food intake

**Fig. 2:**
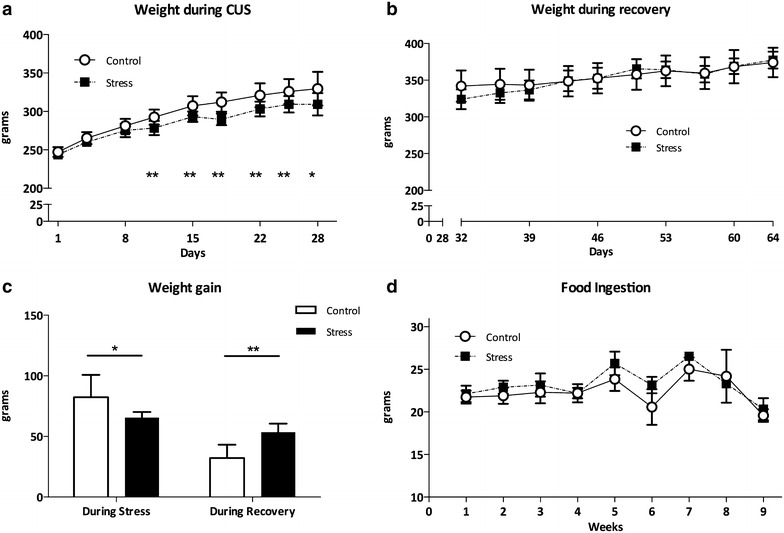
Weight gain and food ingestion. CUS-submitted animals gained less weight during the stress protocol (**a** ANOVA (group), F(1.11) = 10.81; p < 0.05) but gained more weight than controls during the recovery phase (**b** ANOVA, F(1.5) = 0043, p > 0.05 and **c**). These variations occurred without significant variations in food ingestion (**d**). *CTL* controls; *CUS* chronic unpredictable stress. *p < 0.05; **p < 0.01

A significant effect of both ‘time’ and ‘group’ was observed in the weight gain during the period of stress (Fig. 2a; p < 0.05). This means that the CUS-treated group showed lower body weight gain over the 4 weeks of CUS when compared with control animals (Fig. 2c, p < 0.05). During recovery from stress, however, previously stressed animals gained more weight than controls (Fig. 2c, p < 0.05) and the group effect was lost in body weight gain (Fig. 2b; p = 0.88). Interestingly, no significant difference was registered in the total amount of food ingested by control, CUS or post-CUS recovery groups (Fig. 2d).

### Persistent glucose intolerance phenotype induced by stress

**Fig. 3:**
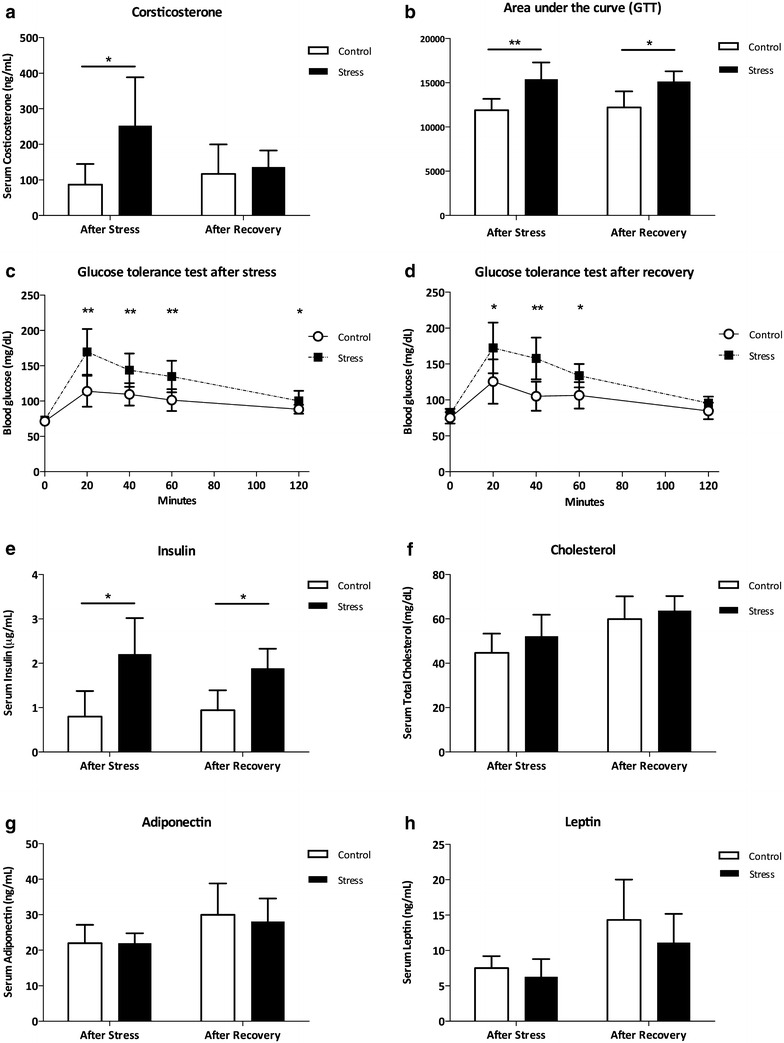
Glucose homeostasis and adipokines in blood. Corticosterone was significantly elevated in animals exposed to CUS but returned to basal levels after recovery (**a**). CUS-submitted animals displayed an glucose intolerance profile that persisted after recovery, characterized by higher levels of glucose in the GTT (**b** area under the curve; **c**, **d** plot at specific timepoints) and hyperinsulinemia (**e**). No differences were found in cholesterol (**f**), leptin (**g**) or adiponectin (**h**) between controls, CUS and CUS after recovery. *AUC* area under the curve; *CTL* controls; *CUS* chronic unpredictable stress. *GTT* glucose tolerance test. *p < 0.05; **p < 0.01

Animals submitted to CUS displayed significantly higher serum levels of corticosterone as compared to control (Fig. 3a; p < 0.05). Following a 5-week stress-free period, corticosterone levels were similar in both CUS-Rec and control groups (Fig. 3a; p > 0.05).

Several parameters were monitored to assess endocrine and glucose homeostasis. A significant effect of both ‘time’ and ‘group’ was observed for the GTT after stress (Fig. 3c: F (1.11) = 27.02; p < 0.05) and after recovery (Fig. 3d: F(1.4) = 8.21; p < 0.05). This means that the CUS-treated animals displayed higher levels of glucose during the GTT, both after stress and after recovery. This result was corroborated by the analysis of the area under the curve (Fig. 3b; p < 0.05).

Consistent with the glucose intolerance profile of CUS-treated animals, insulin levels were significantly higher in CUS *vs.* control animals (Fig. 3e; p < 0.05). Plasma insulin levels were also higher in CUS-Rec when compared with control animals (Fig. 3e; p < 0.05).

Total blood cholesterol levels did not differ between groups (Fig. 3f; p > 0.05). Likewise, as shown in Fig. 3g, serum leptin levels were neither affected in CUS (p > 0.05) nor in the CUS-Rec groups (p > 0.05). Serum levels of adiponectin were also not influenced by any of the treatments (Fig. 2h; p > 0.05).

### Persistent decreased expression of PPARγ in vWAT after CUS

**Fig. 4:**
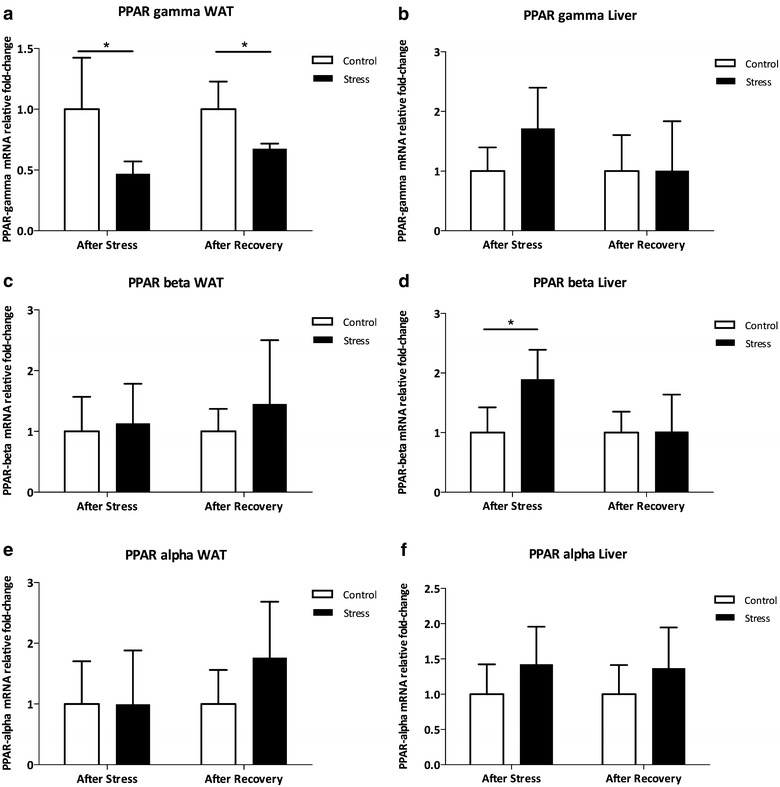
Expression of peroxisome proliferator-activated receptors (*PPAR*). Animals submitted to CUS displayed a lower expression of PPAR-γ mRNA quantified by real-time PCR in the white adipose tissue (vWAT) both after stress and after recovery (**a**). No differences were found in the expression of PPAR-γ mRNA in the liver (**b**). The expression of PPAR-β was similar between CUS and the controls in the vWAT (**c**), while in the liver the expression of PPAR-β was elevated in CUS after stress returning to basal levels after recovery (**d**). No differences were found in the expression of PPAR-α between CUS and controls neither in the vWAT (**e**) nor in the liver (**f**). *CTL* controls; *CUS* chronic unpredictable stress; *PPAR* peroxisome proliferator-activated receptors. *p < 0.05; **p < 0.01

**Fig. 5:**
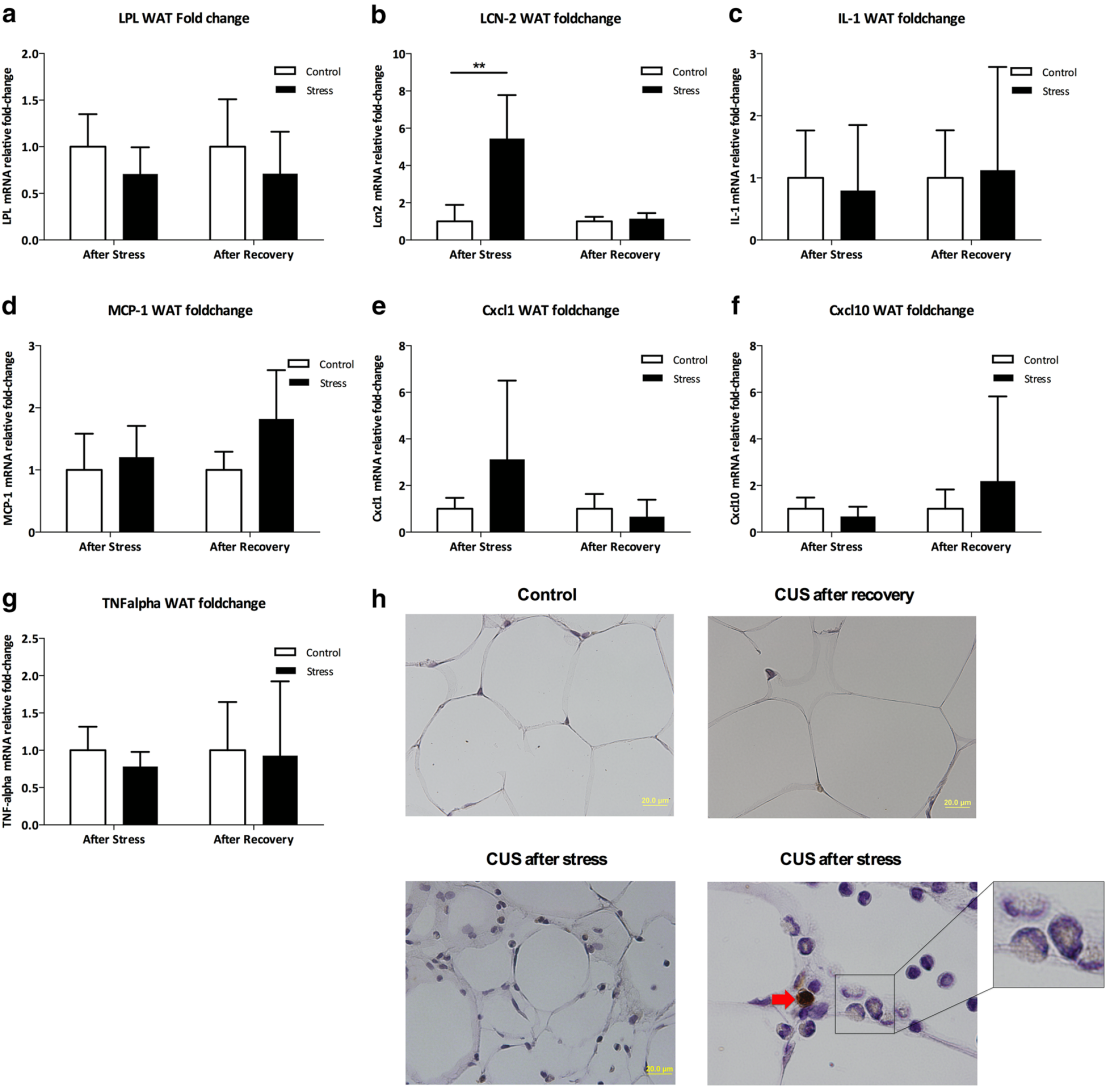
Expressome and Morphologic analyses of the white adipose tissue. The expression of Lcn2 mRNA quantified by real-time PCR was significantly increased in animals submitted to CUS in the white adipose tissue and returned to basal levels after the recovery period (**a**). No differences were found in the expression of TNF-α, IL-1, Mcp-1, Cxcl-1 or Cxcl-10 in vWAT (**b**–**g**). **h** shows the immunohistochemistry of vWAT. It revealed the presence of Lcn2 positive mononuclear inflammatory cells in CUS-submitted animals (**h**), mainly composed by macrophages and monocytes (*inset*); and also the presence of Lcn2 protein-positive inflammatory cells in CUS group after stress (*red arrow*). These findings reverted after recovery. *CUS* chronic unpredictable stress; *Cxcl* chemokine (C-X-C motif) ligand; *IL* interleukin; *Lcn2* lipocalin-2; *LPL* lipoprotein lipase; *Mcp*-1 monocyte chemoattractant protein-1; *TNF*-α tumor necrosis factor-α; *p < 0.05; **p < 0.01

The next task of this study was to characterize the transcription of molecules potentially involved in the pathophysiology of glucose intolerance in the vWAT. A significant decrease in PPAR-γ mRNA levels was observed in vWAT in both CUS (Fig. 4a; p < 0.05) and CUS-Rec (Fig. 4a; p < 0.05). No changes were observed in PPARγ mRNA levels in liver after stress (Fig. 4b; p > 0.05) and after stress recovery (Fig. 4b). CUS did not significantly alter PPAR-β and -α mRNA levels in vWAT (Fig. 4c, f, respectively) but increased PPAR-β expression in the liver (Fig. 4d, p < 0.05) although this effect was reversed after recovery (Fig. 4d; p > 0.05). Expression of PPAR-α mRNA in the liver was neither affected by CUS nor CUS-Rec (Fig. 4f). We also evaluated the transcription of PPAR-γ target genes such as lipoprotein lipase (LPL), whose expression did not display significant differences (Fig. 5a).

### Increased expression of LCN2 in vWAT after CUS

As previously mentioned, vWAT inflammation and the expression of pro-inflammatory cytokines in this tissue have been shown to be involved in the pathophysiology of insulin resistance. As so we quantified the expression of several pro-inflammatory and chemoattractant molecules. Comparison of vWAT from control, CUS and CUS-Rec revealed that, relative to the control condition, CUS resulted in a significant increase in *Lcn2* mRNA levels (Fig. 5b; p < 0.05), and that *Lcn2* expression was restored to control levels after recovery (Fig. 5a; p > 0.05). Immunohistochemical analysis showed the presence of infiltrates of mononuclear inflammatory cells, mainly composed by macrophages and monocytes (Fig. 5h, inset); and also the presence of Lcn2 protein-positive inflammatory cells in vWAT from CUS-treated animals (Fig. 5h). In contrast, Lcn2-positive cells were not detectable in vWAT from CUS-Rec animals (Fig. 5h). In spite of the observation of macrophage infiltration of adipocytes from CUS-exposed animals, this was not accompanied by increased expression of inflammatory (IL-1 and TNFα) or key chemoattractant proteins such as Cxcl-1, Cxcl-10 and *Mcp*-*1* (Fig. 5c–f). Lastly, adipocyte volume and cell density did not differ between the control, CUS and CUS-Rec groups (Table 1).

**Table 1:** Volume and density of the adipocytes in the white adipose tissue

	After stress		After recovery	
	Controls	CUS	Controls	CUS
Volume (×10^5^ μm^3^)	47.8 ± 13.4	45.5 ± 12.7	74.4 ± 13.4	64.1 ± 18.7
Density (×10^−4^)	5.3 ± 1.0	4.8 ± 1.0	3.2 ± 0.8	3.74 ± 1.1

No differences were found in the volume and density of adipocytes between chronic stress and controls both after stress and after recovery

*CUS* chronic unpredictable stress

## Discussion

This study reproduced the phenomenon of stress-induced glucose intolerance, characterized by higher levels of basal insulin and higher levels of glucose during the GTT. This phenotype was accompanied by a decrease in the expression of PPAR-γ, an increase in the expression of *Lcn2* in the vWAT, and an increase in the morning levels of corticosterone. Interestingly, while the expression of *Lcn2* and the levels of corticosterone normalized after recovery, the glucose intolerance phenotype and the lower expression of PPAR-γ prevailed after 5 weeks of recovery from stress.

Previous studies had already examined the relationship between stress and glucose intolerance, using different paradigms of chronic stress and diets [21–29]. To avoid the latter as a confounding factor, we studied the effects of CUS in animals that only had access to regular chow. Interestingly, our CUS-treated rats displayed hyperinsulinemia and glucose intolerance in spite of having less body weight than controls. On contrary to other studies [29], the differences in weight occurred without alterations in food ingestion supporting the idea that stress promotes a hypercatabolic state [25]. Unfortunately, the housing of two animals/cage to avoid the confounding factor of isolation stress [30], precluded the analysis of individual food consumption rates. Moreover, the alterations in weight gain were not accompanied by differences in adipocyte size in the vWAT or differences in the serum levels of leptin, adiponectin or cholesterol. The serum level of these hormones was altered in studies where chronic stress was administered together with energy-rich diets [31]. This highlights the fact that altered glucose intolerance may be the primary mechanism through which stress disrupts metabolism.

Another important observation of our study was that CUS-induced glucose intolerance persisted for 5 weeks after the stress protocol. The long-lasting metabolic effects of CUS were also shown in the adult offspring of stressed pregnant females [32]. Importantly, following recovery from CUS, glucocorticoid levels were restored to those found in control animals as well as the expression of *Lcn2* in the vWAT. Thus, the direct effect of glucocorticoids on the levels of glucose cannot be the explanation for the glucose intolerance found in CUS-Rec. At this level PPARs may play a crucial role.

As previously mentioned, PPAR-γ up-regulates the transcription of genes mainly involved in fatty acid metabolism and triglyceride storage, promoting adipogenesis and lipids uptake to the adipose tissue [17, 18]. These events decrease the serum levels of free fatty acids and induce a lipid repartitioning from the skeletal muscle and liver to the adipose tissue, thus eliminating the deleterious effects of lipids on insulin signaling [17, 18]. Matsusue and colleagues showed that, in diabetic obese mice with liver-specific PPAR-γ deletion, thiazolidinediones (a full PPAR-γ agonist) remained effective in lowering glucose levels, contradicting the hypothesis that liver PPAR-γ was the responsible for the insulin sensitization effects of these drugs [33]. Accordingly, our results show that CUS caused a selective downregulation of PPAR-γ mRNA expression in vWAT that persisted after recovery. This indicates that vWAT is an important target tissue of stress and that the disruption of PPAR-γ signaling may be responsible for the perpetuation of glucose intolerance after CUS recovery. PPAR-γ is mainly expressed in the adipose tissue and only in a small amount in the liver (in our data, control animals displayed an expression of PPAR-γ in the vWAT approximately 5 times superior to the expression in the liver). This may justify the selective downregulation of PPAR-γ in the adipose tissue. Interestingly, in cardiomyocytes the regulation of PPAR signaling was also shown to be involved in the pathophysiology of diabetic cardiomyopathy [34]. The evidence concerning modulation of PPAR-γ expression by glucocorticoids is still very scarce but it is of notice that few experiments in adipocytes show that PPAR-γ is responsive to glucocorticoids [35, 36]. This supports the idea that glucocorticoid-induced disruption of PPAR-γ signaling may underlie stress-induced glucose intolerance. We did not find statistically significant differences in the expression of LPL (which is a target gene of PPAR-γ) among groups. This may be explained by the fact that LPL is regulated by other mediators that not only PPAR-γ. Beside its effects on lipid metabolism, PPAR-γ activation also exerts anti-inflammatory activity by suppressing the production of inflammatory mediators as *IL*-*6*, plasminogen activator inhibitor-1, *Mcp*-*1* and TNFα [37].

In recent years, the importance of adipose tissue inflammation has gained increasing attention, as a result of studies showing a clear association between inflammation and insulin resistance [38]. In general, pro-inflammatory cytokines produced in the adipose tissue promote insulin resistance mainly by promoting lipolysis and disruption of the insulin and leptin signaling in skeletal muscle and liver [39]. Surprisingly we did not observe differences in the expression of pro-inflammatory or chemoattractant cytokines after CUS (as previously described by others [27, 40]). Instead, we showed that CUS elicited only a very significant overexpression of *Lcn2*, which is considered an acute-phase response protein [41]. This was accompanied by the appearance of Lcn2 positive cells infiltrating the adipose tissue. From a clinical perspective *Lcn2* has been validated as a useful marker of metabolic syndrome [42] and has been implicated in the pathophysiology of insulin resistance by antagonizing the detrimental effects of inflammatory molecules (in particular TNFα) on the metabolism of adipocytes and macrophages, mainly through the modulation of PPAR-γ expression [43]. Lcn2 was also shown to be a selective modulator of PPAR-γ activation, being necessary to its full activation by thiazolidinediones, due to their interaction at the level of the recruitment of coactivators/corepressors [41]. In spite of these observations, which were partially derived from in vitro studies, Lcn2 was found to be overexpressed in the adipose tissue of obese animals [43]. However, conflicting results have been described regarding (increased or decreased) glucose intolerance in Lcn2 knockout mice [44, 45]. Together with human studies, the general consensus is that Lcn2 is overexpressed during insulin resistance. Of note, this Lcn2 overexpression may be driven by glucocorticoids since Lcn2 has a glucocorticoid response element [46]. Interestingly, elevated adipose tissue Lcn2 levels can be normalized by the insulin-sensitizing drug rosiglitazone reinforcing the interconnection between PPAR-γ and this molecule [43]. In spite of this evidence the fact is that the levels of PPAR-γ remain low while the levels of Lcn2 return to normal after recovery. This suggests that there are other mechanisms regulating the expression of Lcn2 and PPAR-γ. Given the presence of Lcn2 positive infiltrating cells after stress we may speculate that the origin of the overexpression of Lcn2 is not coming from the adipocyte itself but rather from macrophages infiltrating the adipose tissue. Despite these results it is still a matter of debate whether this Lcn2 overexpression is beneficial or detrimental to the glucose intolerance phenotype.

In this study, we did not explore other potential pathways through which an association between stress and glucose intolerance can be established. Specifically, several studies suggest that the disruption of nitric oxide pathway [47, 48] and oxidative stress [40] may also contribute to stress-related glucose intolerance and vascular dysfunction. Another limitation of this study was the lack of a more thoroughly analysis of the insulin signaling to determine if the glucose intolerance reported here is associated with insulin resistance.

## Conclusion

In summary, our experimental work shows that the metabolic effects of chronic stress persist after a period of recovery and that the glucose intolerance triggered by chronic stress is associated with a decrease in the expression of PPAR-γ and a transient overexpression of Lcn2 in the vWAT. The characterization of the mechanistic pathways of stress-related disorders will significantly contribute to the design of appropriate interventions for patients suffering from these conditions (e.g. depression or anxiety). Concerning the glucose intolerance we believe that in a near future, new experiments using pharmacological approaches (e.g. thiazolidinediones) will shed more light into this topic.

## Abbreviations

ANOVA: analysis of variance
CUS: chronic unpredictable stress
Cxcl: chemokine (C-X-C motif) ligand
GTT: glucose tolerance test
Hprt: hypoxanthine guanine phosphoribosyl transferase
IL: interleukin
Lcn2: lipocalin-2
LPL: lipoprotein lipase
Mcp-1: monocyte chemoattractant protein-1
PPAR: proliferation-activated receptor
TNF-α: tumor necrosis factor-α
vWAT: white adipose tissue
